# Gynecological, reproductive and sexual outcomes after uterine artery embolization for post-partum haemorrage

**DOI:** 10.1038/s41598-020-80821-0

**Published:** 2021-01-12

**Authors:** Béatrice Eggel, Maude Bernasconi, Thibaud Quibel, Antje Horsch, Yvan Vial, Alban Denys, David Baud

**Affiliations:** 1 Materno-Fetal and Obstetrics Research Unit, Department “Femme-Mère-Enfant”–“Woman–Mother–Child”, University of Lausanne and Lausanne University Hospital, 1011 Lausanne, Switzerland; 2 Institute of Higher Education in Healthcare Research, University of Lausanne and Lausanne University Hospital, 1011 Lausanne, Switzerland; 3 Neonatology Service, Department Woman–Mother–Child, University of Lausanne and Lausanne University Hospital, 1011 Lausanne, Switzerland; 4 Department of Radiology, University of Lausanne and Lausanne University Hospital, 1011 Lausanne, Switzerland; 5 Obstetric Service, Department “Femme-Mère-Enfant”–“Woman–Mother–Child”, Centre Hospitalier Universitaire Vaudois (CHUV), University of Lausanne and Lausanne University Hospital, 1011 Lausanne, Switzerland

**Keywords:** Anatomy, Diseases, Risk factors

## Abstract

In this case control study, long-term gynecological, reproductive and sexual outcomes after uterine artery embolization (UAE) for postpartum hemorrhage (PPH) were evaluated. The study was performed in a single referral hospital for PPH in Lausanne from 2003 to 2013. Each woman whose delivery was complicated by PPH and treated by UAE was included, and compared to a control group of women whose delivery was uncomplicated. Cases were matched by maternal age, parity, ethnicity, year and mode of delivery, birth weight and gestational age in a 1–3 ratio. A total of 77 patients treated by UAE for PPH were identified in our obstetrical database. Among them, 63 were included and compared to 189 matched patients (no PPH). The mean interval time between UAE and this study was 8.1 years. Time to menstrual cycle recovery after delivery (3.9 vs 5.6 months, p = 0.66), spotting (7.9% vs 7.2%, p = 0.49), dysmenorrhea (25.4% vs 22.2%, p = 0.60) and amenorrhea (14.3% vs 12.2%, p = 0.66) were similar between the two groups. There was no difference in the FSFI score between the groups (23.2 ± 0.6 vs 23.8 ± 0.4; p = 0.41). However, the interval time to subsequent pregnancy was longer for patients after UAE than the control group (35 vs 18 months, p = 0.002). In case of pregnancy desire, the success rate was lower after UAE compared to controls (55% vs 93.5%, p < 0.001). The rate of PPH was higher in those with previous PPH (6.6% vs 36.4%, p = 0.010). Patients treated by UAE for PPH did not report higher rates of gynecological symptoms or sexual dysfunction compared to patients with uneventful deliveries. The inter-pregnancy interval was increased and the success rate was reduced. In subsequent pregnancies, a higher rate of PPH was observed in those that underwent UAE.

## Introduction

Postpartum hemorrhage (PPH) remains one of the major causes of maternal morbidity and mortality worldwide. The World Health Organization estimates that postpartum hemorrhage accounts for 25% of all maternal deaths^1^. Most cases of PPH are resolved by fundal massage and uterotonic medications, but major bleeding may require second line procedures, such as pelvic artery embolization, uterine tamponade or peripartum hysterectomy as lifesaving options^2^. The goal of UAE in the context of PPH is to occlude temporarily blood vessels with an absorbable material to control bleeding^3^. Even though arterial embolization requires equipment and an interventional radiologist, some national guidelines (such in France) recommend embolization as a therapy after failure of medical treatment for PPH in cases of stable maternal haemodynamics. Indeed, in a large retrospective study including 251 patients, Lee et al*.* found that UAE was successful in arresting the bleeding in 86.5% of the cases. However, temporary blood vessel occlusion may induce local necrosis and inflammatory reactions, with potential subsequent adverse effects on the endometrium and ovaries, leading to a disturbance of the menstrual cycle and fertility^4,5^. Cases of partial or total uterine necrosis after UAE for PPH have also been described^6,7^. Nevertheless, it is considered to be a safe and effective alternative to surgery, but there are still concerns about the long term consequences of UAE on women’s health. A recent systematic review has described gynecological and obstetrical adverse outcomes after UAE for PPH, but most of the studies were limited in power, based on short term evaluations or not reporting a control group for comparisons. More, there is no study reporting sexual function after UAE with validated questionnaire^8^.

The aim of the present study was to evaluate long-term gynecological, sexual and reproductive outcomes for patients who underwent UAE for PPH in comparison to a control group of patients without PPH.

## Materials and methods

This case control study was designed using our obstetrical database at the University Maternity Hospital, Lausanne, Switzerland, which is a referral hospital in the region of the French part of Switzerland for PPH. All data were collected prospectively at the time of delivery by the obstetrician or midwife. We previously cross-checked our database, with confirmation of congruent data in > 98% of cases^9^.

For each delivery in our institution, graduated collector bags were placed just after birth, left in place at least for 15 min, and then until the birth attendant judged that bleeding had stopped.

Management of PPH followed national guidelines with manual exploration of the uterus, visual assessment of genital tract, bladder indwelling catheter, uterine massage and use of a uterotonic (syntocinon followed by sulprostone). Blood transfusions were performed when there was clinical evidence of inadequate oxygen-carrying capacity or an haemoglobin concentration < 70 g/l. Fresh frozen plasma was transfused in the presence of consumption coagulopathy and persistent bleeding. If these local measures were unlikely to control the hemorrhage, UAE was then considered both after vaginal or caesarean delivery.

Amount of blood loss was recorded in the medical file. We used classical definition of PPH (blood loss of 500 ml or more after delivery) to identify first eligible women in the study, and then patients who were treated with UAE were included. Cases were compared to a group control, which included deliveries that were not complicated with PPH, and thus who did not experience UAE. Cases were matched in a 1 to 3 ratio by maternal age, parity, ethnicity, year and mode of delivery, birth weight and gestational age. Deliveries that occurred at a gestational age less than 24 weeks of gestation or which requested peripartum-hysterectomy were excluded from the database.

The current home address of these women was identified using the telephone directory. One of the authors attempted to contact all the patients directly by telephone. Patients were informed about the study and included after their consent. Current social, demographic, and physical characteristics were registered.

Patients were asked about gynecological symptoms, such as time to return to menstrual cycles, menstrual characteristics (quantity, dysmenorrhea), modification of cycles, contraception, pelvic pain and potential gynecologic interventions after the index pregnancy. Patients were also questioned about desire for subsequent pregnancy and attempts to conceive. Data of the subsequent following pregnancy were recorded: date of pregnancy, pregnancy outcome (miscarriage/livebirth), mode of delivery, gestational age at birth, infant birth weight, recurrence of PPH and need for blood product transfusion. Regarding the sexual dysfunction, the Female Sexual Function Index (FSFI) questionnaire was sent to patients. This Index has a high reliability and validity and is validated in French^10,11^. This multidimensional score combines 19 questions in six subscales (desire, arousal, lubrication, orgasm, satisfaction, and pain). The score ranges from 2 to 36, with high scores representing high sexual activity and satisfaction and low scores signifying sexual difficulties or little sexual activity. Both groups received the same questionnaires by phone and by mail. The study was approved by the Ethical Committee of the University and Hospital Lausanne, Switzerland (protocol 55/13; date of approval 07.16.2013). We confirm that all methods were performed in accordance with the relevant guidelines and regulations, and informed consent was obtained from all participants.

### Statistical analyses

Demographic data and patient responses were compared between the case and control groups using the Pearson χ^2^ test (or the Fisher’s exact test when indicated) for categorical variables. For continuous variables, means were compared by the Student's t test. Statistical analyses were performed using STATA-15 (Stata Corporation, College Station, USA).

## Results

From 2003 to 2013, 27,344 women delivered in our institution. Among them, 875 (3.2%) had a PPH and 77 (0.2%) underwent UAE. A total of 12 women were lost to follow-up and 2 declined to participate. The 63 remaining cases were compared to 189 women without PPH who were matched by age, parity, ethnicity, year and mode of delivery, birth weight and gestational age. Sociodemographic and index delivery characteristics of women with and without UAE were similar (Table 1). The mean interval between the index delivery and response to the questionnaires was 8.1 years [95% CI 7.8–8.4].

**Table 1:** socio-demographic characteristics at the index delivery (SD: standard deveiation; min: minutes).

	Controls	Cases	*p* value
	Without embolization (n = 189)	With embolization (n = 63)	
Age (years + SD)	32.6 ± 4.3	32.9 ± 4.7	0.539
Gestational age at birth (weeks + SD)	38.2 ± 2.8	37.8 ± 2.9	0.594
Birth weight at index pregancy (gr + SD)	3076 ± 714	2874 ± 825	0.136
Arterial pH < 7.1	5 (2.7%)	0 (0%)	0.192
Apgar < 7at 5 min	8 (4.2%)	2 (3.2%)	0.709
**Parity**			
1	46 (24.4%)	23 (36.5%)	0.155
2	87 (46.0%)	26 (41.3%)	
>3	56 (29.6%)	14 (22.2%)	
**Nationality**			
Swiss citizen	86 (45.5%)	23 (36.5%)	0.212
Non swiss	103 (54.5%)	40 (63.5%)	

### Gynecological outcomes

Time to return of menstrual cycles in patients with UAE was shorter than those without UAE (Table 2), without reaching significance (3.9 versus 5.2 months; p = 0.066). Menstrual cycle disturbances such as amenorrhea, dysmenorrhea, and modification of cycles were similar between both groups. About half of the patients were using hormonal contraception at the time of the questionnaire. The rate of any subsequent gynecological surgery after the index delivery was similar between both groups.

**Table 2:** Gynecological outcomes (95% CI; 95% confidence intervals).

	Controls	Cases	*p* value
	Without embolization n = 189 (%)	With embolization n = 63 (%)	
Menses after delivery, months [95% CI]	5,7 [4.6–6.9]	3.9 [2.9–4.9]	0.066
Hormonal contraception	101 (53.4%)	30 (47.6%)	0.423
Gynecological surgery	13 (6.9%)	6 (9.5%)	0.491
**Menstrual disturbance**			
Amenorrhea	23 (12.2%)	9 (14.3%)	0.662
Dysmenorrhea	42 (22.2%)	16 (25.4%)	0.604
Spotting	14 (7.4%)	5 (7.9%)	0.496
Modified cycle	80 (42.3%)	27 (42.9%)	0.866

### Sexual dysfunction

There was no difference in the mean total score of the FSFI between patients with and without UAE for PPH. More, for each subscale item, the score was similar, except for “pain during vaginal penetration about half of the time or more” (21.1% in the UAE group vs 7.8% in the control group, p = 0.027). Table 3 sums up sexual dysfunction with the FFSI score.

**Table 3:** Sexual dysfunction.

FSFI questionnaire	Patients	Patients	p value
	Without embolization % (n = 104)	With embolization % (n = 38)	
Sexual desire half of the time or less	17.5	27.8	0.185
Low level of sexual desire	22.3	27.0	0.564
**Score "DESIRE" (1.2–6)**	4.0 ± 0.1	3.9 ± 0.2	0.560
Excitation during sexual activity half of the time or less	53.9	63.9	0.300
Low level of excitation during sexual activity	42.2	54.1	0.213
Low confidence about becoming sexually excited during sexual activity	25.5	24.3	0.889
Satisfied with excitation during sexual activity less than half of the time	61.8	59.5	0.805
**Score "EXCITATION" (0–6)**	3.4 ± 0.1	3.3 ± 0.2	0.825
Lubrication during sexual activity less than half of the time	55.9	50.0	0.534
Difficulty becoming lubricated during sexual activity	7.9	10.5	0.626
Maintain lubrication until completion of sexual activity less than half of the time	61.8	56.8	0.594
Difficulty maintaining lubrication until completion of sexual activity 7.5	12.0	13.2	0.853
**Score "LUBRICATION" (0–6)**	4.4 ± 0.1	4.4 ± 0.8	0.504
Reach orgasm less than half of the time	24.5	40.5	0.065
Reaching orgasm difficult	12.9	8.1	0.439
Moderatly dissatisfied with ability to reach orgasm	56.9	59.5	0.784
**Score "ORGASM" (0–6)**	4.0 ± 0.1	3.9 ± 0.1	0.696
Moderatly satisfied with amount of emotional closeness during sexual activity	55.9	62.2	0.508
Moderatly dissatisfied about the sexual relationship	58.4	59.5	0.912
Moderatly dissatisfied about overall sexual life	50.0	59.5	0.324
**Score "SATISFACTION" (0–6)**	2.7 ± 0.1	2.9 ± 0.2	0.694
Pain during vaginal penetration about half of the time or more	7.8	21.1	0.027
Pain following vaginal penetration more than half of the time	2.9	5.3	0.503
High level of pain during or following vaginal penetration	6.8	15.8	0.101
**Score "PAIN" (0–6)**	5.1 ± 0.1	5.0 ± 0.2	0.673
**Score total (2–36)**	23.8 ± 0.4	23.2 ± 0.6	0.412

### Reproductive outcomes

The rate of women who planned to have another pregnancy after the index pregnancy (Table 4) was similar between the two groups (33.3% in the UAE group vs 41.8% in the group control, p = 0.234). The rate of spontaneous pregnancy for the subsequent pregnancy was not different (100% in the UAE group vs 91.1% in the control group, p = 0.206).

**Table 4:** Desire for and fear of subsequent pregnancy.

	Controls	Cases	*p* value
	Without embolization n = 189 (%)	With embolization n = 63 (%)	
Desire for subsequent pregnancy	77 (41.8%)	20 (33.3%)	0.234
Fear as reason for pregnancy avoidance	9/112 (8.2%)	16/43 (38.1%)	< 0.001
Unplanned pregnancy	7 (3.7%)	2 (3.2%)	0.845

**Figure 1:**
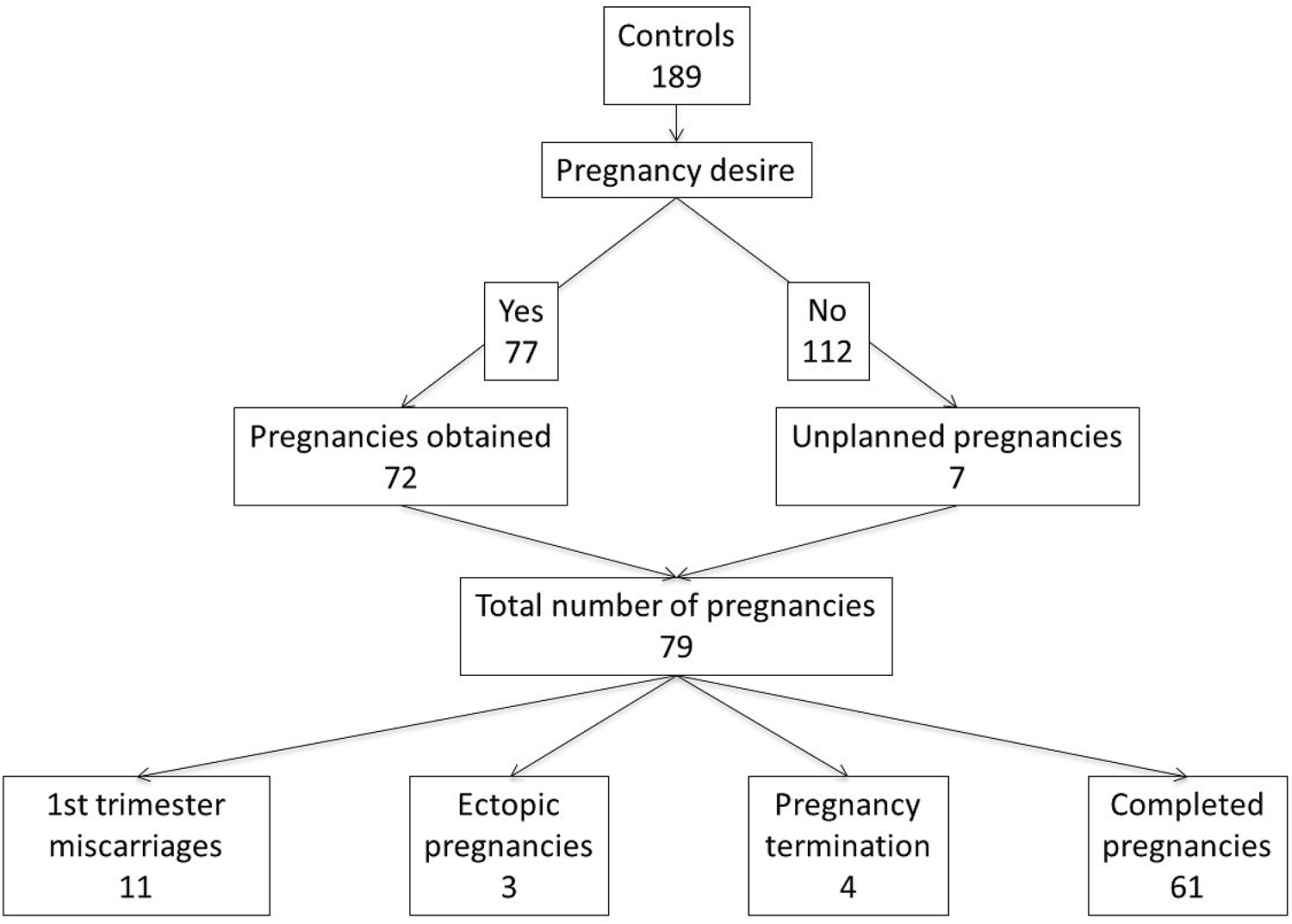
pregnancy desire and pregnancy outcomes, control patients.

**Figure 2:**
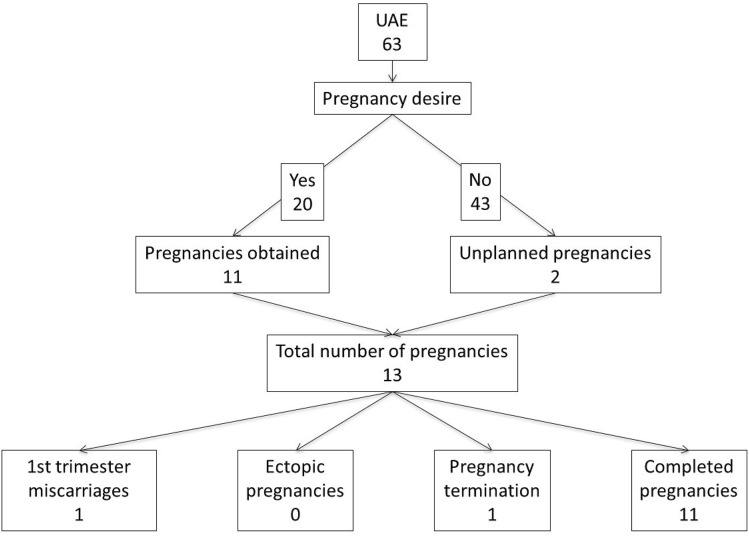
pregnancy desire and pregnancy outcomes of embolized patients (UAE).

Among patients who pursued another pregnancy (Figs. 1 and 2 should be side by side to be able to be compared), those with UAE were significantly less successful than those without UAE (55.0% versus 93.5%, p < 0.001, Table 5). Additionally, a significantly higher time to conception was present in those conceiving after UAE than controls (35 versus 18 months, p = 0.002). The arguments put forward by patients to justify this delay were mainly the fear of a recurrence of PPH. No patient who underwent UAE required an assisted reproductive procedure. There was no significant difference in the rate of unplanned pregnancies between the 2 groups.

**Table 5:** Fertility outcomes among patients with interest in subsequent pregnancy (95% CI: 95% confidence intervals).

	Controls	Cases	*p* value
	Without embolization n = 189 (%)	With embolization n = 63 (%)	
Interval to next pregnancy, months [95% CI]	18 [14–22]	35 [21–50]	0.002
Pregnancy obtained	72/77 (93.5%)	11/20 (55%)	< 0.001
Pregancy desired but not attempted at the time of the questionnaire	1/77 (1.3%)	7/20 (35%)	< 0.001
Secondary infertility	4/77 (5.2%)	2/20 (10%)	0.427
Infertility investigation	11/77 (14.4%)	0/20 (0%)	0.180
Spontaneous pregnancies	72/79 (91.1%)	13/13(100%)	0.206

Regarding subsequent pregnancy, the rates of spontaneous miscarriages, terminations of pregnancy, and ectopic pregnancies were similar between patients with and without UAE (Table 6).

**Table 6:** Subsequent pregnancy outcomes (for patients who became pregnant after the index pregnancy). Only the first pregnancy after the index pregnancy is described below.

	Controls	Cases	*p* value
	Without embolization n=189 (%)	With embolization n=63 (%)	
First trimester miscarriage	11 (13.9%)	1 (7.7%)	0.79
Pregnancy termination	4 (4.3%)	1 (7.7%)	1
Extra-uterine pregnancy	3 (3.8%)	0 (0%)	0.575
Spontaneous vaginal birth	40/61 (65.6%)	4/11 (36.4%)	0.67
PPH	4/61 (6.6%)	4/11 (36.4%)	0.015
Birth weight of 1st pregnancy after the index pregnancy (gr + SD)	3224 + 602	3083 + 453	0.461

All the remaining pregnancies delivered at  > 37 weeks gestations. The rate of cesarean section was double in patients with previous UAE compared to controls, without reaching statistical significance (p = 0.67). The rate of PPH was significantly higher in those with previous UAE compared to the controls (36.4% versus 6.6%, p = 0.015).

Birth weight and rate of intra-uterine growth restrictions (IUGR) were similar between both groups. All 72 children were born healthy.

## Discussion

### Main findings

This study confirmed the safety of uterine arterial embolization, as it did not find long term consequences of common gynecological symptoms and sexual functions. However, women with a previous UAE should be appropriately counseled for the risk of recurrence of PPH in a subsequent pregnancy.

### Strength and limitations

Many strengths should be underlined. The high rate of eligible women who were included is undoubtfully a strength, notably after a long-time frame, over which outcomes were examined other than obstetric outcomes making possible a large sample size. Searching for associations between an exposition and an adverse outcome is always challenging, and matching the two groups with several well-known cofounders for PPH as maternal age, mode of delivery, gestational age of delivery remains an accepted method to answer the question. Nevertheless, some cofounders notably for PPH were not taken into account, as history of PPH, use of oxytocin during labor, and caution should be taken in the interpretation of the results. Another limitation is the control group. In order to conclude that the reduction in pregnancy rate is due to UAE, it would be necessary to compare with and without UAE implementation in the population with previous PPH. Indeed, one could also argue that the best controls should be women whose delivery was complicated by PPH and who were not treated with UAE. However, our study did not reveal any difference in most of the outcomes examined, except for the recurrence of PPH. We can therefore assume that UAE is a safe procedure that does not affect occurrence of gynecological symptom or sexual dysfunction compared to women whose delivery was uncomplicated by PPH.

Another limitation of our study, as in many studies evaluating long-term maternal outcomes, was the potential for recall bias, as mentioned above. Evaluation by a phone interview and questionnaires of an event that occurred 8 years ago on average can be subjective and depends on the accuracy of patient memory. Finally, we did not have an evaluation of baseline symptoms prior to the index delivery. It was thus impossible to know whether gynecological symptoms or sexual dysfunction were present prior to the index delivery or whether symptoms were attributable to the UAE.

### Interpretation

UAE plays a major role in the current management of PPH that fails to respond to other conservative treatments. While its short-term benefit is well known (avoid hysterectomy and prevent potential maternal death), long-term clinical and psychological outcomes of UAE have been poorly investigated. In addition to the long-term sequelae induced by psychological stress (sexual dysfunction, fear of another pregnancy), UAE may induce transient ischemia of the uterus and potentially alter the patients’ future menstrual cycles and fertility. The passage of embolization particles into utero-ovarian anastomoses or ovarian arteries originating from the uterine arteries may induce endometrial ischemia altering the implantation success of future fertilized eggs or provoke early menopause.

The present study shows that the timing and characteristics of menses and sexuality were not affected by management of a prior PPH by UAE. The traumatic experience for the patient, however, lowered interest in a subsequent pregnancy. In those patients that underwent UAE and desired a subsequent pregnancy, the success rate was lower and a longer inter-pregnancy interval was observed compared to controls without PPH. The frequency of miscarriage and IUGR were similar between both groups. Cesarean section and PPH rates were higher for patients with previous UAE for PPH than controls.

### Gynecological outcomes

We, and others (reviewed in^8^) did not identify any change in menstrual cycles or gynecological symptoms after UAE. The return of menses was longer in the controls than in patients with UAE, although not significant. After PPH, women are less likely to initiate and sustain exclusive breastfeeding^12^, which may explain this difference, as most mothers who pursue exclusive breastfeeding are amenorrheic for 3–6 months.

The rate of menstrual cycle disturbances described in the present study was similar to those described by others. Sentilhes et al.^13^ identified a 10.3% risk of synechia diagnosed by hysteroscopy after UAE. In our study, data regarding hysteroscopies was not collected and synechia could thus not be excluded in patients with amenorrhea or secondary infertility.

Sexual function following UAE can theoretically be affected by two mechanisms. First, entry of embolization particles into cervical or vaginal anastomoses might induce local ischemia and thus dyspareunia, lack of lubrification, and denervation. Secondly, psychological and environmental factors can also affect sexual function. A life-threatening event could affect the libido of both the patient and partner. As summarized in the Soro et al*.* review, few studies with non-validated questionnaires have evaluated sexual function after UAE and reported inconsistent results. Our evaluation using the FSFI questionnaire did not demonstrate major difference between patients with and without UAE, in particular for lubrication or dyspareunia. Libido, which is particularly dependent on psychological and environmental factors, was not influenced by the events in the index pregnancy.

### Reproductive outcomes

According to Hardeman et al., the fertility of patients after UAE is lower than the general population, without being statistically significant in their study^4^. Our study confirms these results with a similar secondary infertility rate for patients with UAE compared to controls. Additionally, the time to achieve pregnancy is almost doubled for patients with UAE, as also described by Capmas et al.^14^. While the theory of an unfavorable endometrium post-UAE might explain this difference, the psychological impact of the PPH is more likely the key factor for this delay.

Of the 20 patients who planed a subsequent pregnancy, 38% had not yet attempted to achieve a pregnancy by the time of our present study. This mirrors the results presented by Sentilhes et al.^13^. Our study highlights the underlying reason behind this prolonged interval. Patients who are undecided, are delaying conception and those that have opted against a subsequent pregnancy regularly discuss fear of PPH recurrence. These results are in line with studies showing that following traumatic childbirth, women with higher levels of posttraumatic stress disorder (PTSD) symptoms at six weeks postpartum show an increased likelihood of deciding not to have further children; and women after a traumatic birth who do embark on a subsequent pregnancy report delaying this longer than those without such an experience^15^. Indeed, a PPH meets criteria for a traumatic stressor^16^ and it is thus likely, that some, if not many participants, perceived their childbirth as traumatic, which is an important risk factor for postpartum PTSD^17^.

None of the patients with UAE underwent fertility treatment, whereas 14.4% of those in the control group pursuing another pregnancy underwent a fertility treatment (half required assistance for their index pregnancy). Again, “fear” and having experienced their childbirth as traumatic may explain this difference, as those with a past history of requiring UAE for PPH may want to avoid both another PPH as well as any medically related situation.

In our study, we observed a significant difference in the success of achieving a subsequent desired pregnancy between cases and controls. Hardman et al*.*, although lacking significance, made similar observations. The psychological impact of PPH is likely underestimated and plays a prominent role in the fertility rate our patients.

Our results support the hypothesis that UAE does not affect future uteroplacental exchange and thus the rates of miscarriage, IUGR and preterm birth. However, a previous episode of PPH is a risk factor for its recurrence regardless of its initial management^18^, as also verified in our study. However, it is unclear whether the risk of recurrence of PPH is more related to history of PPH or to PPH treated with UAE. More than one third of the patients in this study with PPH and treated with UAE had a recurrence, which is more than the 19.2% described by Soro et al.^8^. Recall bias cannot be excluded in our study, as information regarding subsequent pregnancies was obtained via patient questionnaires without access to the official medical records.

In conclusion, our study confirms the safety of UAE for PPH. The long-term effects in women who have been treated with UAE for PPH seem limited. Since subsequent pregnancy outcomes after UAE were similar to the control group, patients can be reassured during counselling about future pregnancies. Recurrence of PPH, however, is increased and subsequent deliveries should be actively managed accordingly. The psychological impact of PPH should not be underestimated, and warrants further investigations, as performed in the second part of this study. In the management of patients following a UAE, early identification of those experiencing high levels of distress and recommendation of psychological counseling or other appropriate interventions should be strongly considered.

## Abbreviations

UAE: Uterine artery embolization
PPH: Postpartum hemorrhage
